# The Optimization of Demulsification Using Composite Fatty Acids in Aqueous Enzymatic Extraction and the Changes of the Emulsion Stability During Demulsification

**DOI:** 10.3390/foods14050749

**Published:** 2025-02-22

**Authors:** Zhihua Shao, Xiangrui Kong, Hanxiang Yang, Yiyang Zhang, Chenxian Yang, Fusheng Chen, Zikun Wang, Jiaxun Chen, Tingwei Zhu, Ying Xin, Yu Chen

**Affiliations:** College of Food Science and Engineering, Henan University of Technology, Zhengzhou 450001, China; 13164395903@163.com (Z.S.); 13383798253@163.com (X.K.); 13103840110@163.com (H.Y.); zy2856458004@163.com (Y.Z.); 19714519987@163.com (Z.W.); 19233832191@163.com (J.C.); zhutingwei@haut.edu.cn (T.Z.); huilier323@126.com (Y.X.); chenyu@haut.edu.cn (Y.C.)

**Keywords:** aqueous enzymatic extraction, composite heptanoic and octanoic acid, demulsification, emulsion stability

## Abstract

Aqueous enzymatic extraction (AEE) can simultaneously separate oil and protein. However, a stable O/W emulsion is present in the AEE process, which is not favorable for extracting oils. This study optimized the use of heptanoic and octanoic acids for demulsification in aqueous enzymatic extraction. The optimal condition for demulsification, including a fatty acid ratio of 1:3 (heptanoic acid to octanoic acid) with 1.00% addition, a reaction time of 40 min, a temperature of 70 °C, and a solid-to-liquid ratio of 1:5, resulted in a demulsification rate of 97.95% ± 0.03%. After demulsification, the particle size of the peanut emulsion increased, while the absolute potential value and conductivity decreased. The type and content of proteins decreased, and the tertiary structure also changed, with tryptophan residues buried within the proteins, shifting the system from a polar to nonpolar environment. The microstructure of the emulsion changed and the emulsion transformed into W/O. To summarize, composite fatty acid had a significant effect on the demulsification of emulsion.

## 1. Introduction

Peanuts are widely cultivated and their annual production is very high worldwide. Peanuts are one of the major oilseeds and cash crops in China [1]. According to latest report released by the state, the cultivated area of peanuts is 4.8 million hectares, with an annual output of 19 million tons in China. Peanut planting area accounts for 23% of the country’s total oilseed planting area and contributes to 50% of the annual output. Peanuts are rich in fats and proteins, with contents of 20–28% and 40–58%, respectively. They contain eight essential amino acids required by the human body [2]. Therefore, the utilization value of peanuts needs to be increased for global resource utilization.

Aqueous enzymatic extraction (AEE) is a new eco-friendly oil extraction technology. It uses water as a solvent to separate oils and proteins in oilseeds, and cell structures are degraded and destroyed to release oils and proteins by using enzymes. Additionally, it can efficiently recover other valuable components, such as polysaccharides in oilseeds [3,4,5]. Liu et al. [6] extracted castor seed oil by AEE and the results showed that neutral protease was the optimal enzyme for extracting castor seed oil, with a high extraction rate of 77.53% under the optimum condition. Xu et al. [7] extracted rice bran oil with AEE and reported that the quality of rice bran oil was improved by AEE. Sorita et al. [8] performed AEE of macauba pulp oil (MPO) using five commercial enzyme pools. AEE has several advantages over conventional oil extraction, including a simplified process, an eco-friendly approach, food safety, higher product utilization, and greater oil and protein yields. Thus, this approach provides a new pathway with good application potential for simultaneously separating oils and proteins in oilseeds [9]. Although AEE has many advantages, stirring and centrifugation cause a stable emulsion to form. Proteins and phospholipids act as emulsifiers and prevent oil droplets from coalescing, thus reducing the oil yield. This severely limits the potential for AEE in industry [10]. Therefore, demulsification is necessary to increase oil yield.

Aqueous enzymatic extraction demulsification (AEED) methods can be divided into four main categories: physical, chemical, bioenzymatic, and composite methods. Physical demulsification methods include sedimentation and separation demulsification, ultrasonic demulsification, microwave demulsification, and membrane demulsification [11,12,13,14]. The sedimentation and separation methods of demulsification are less efficient and time-consuming [15]. No suitable industrial equipment is available for ultrasonic demulsification in the oil recovery field [16]. The microwave demulsification method has a limited and uneven effective demulsification area, along with high equipment and operating costs [12]. The cost of membrane demulsification is very high, and the process may cause environmental pollution [13]. The chemical demulsification method primarily relies on the use of chemical agents to destabilize the emulsion, thereby achieving demulsification [17]. So the chemical demulsification leads to the formation of certain byproducts that are harmful to the environment [18]. Biodemulsification is a method of destabilizing emulsions through the production of specific substances during the metabolic processes of microorganisms with particular characteristics [19]. Biological demulsifiers are divided into cell-bound demulsifiers, extracellular metabolic demulsifiers, and enzymes [20]. The advantages of biological demulsification include high efficiency, low cost, low toxicity, ease of degradation, and the presence of various biological structures [21]. Moreover, biological demulsification technology has the advantages of safe composition, a wide range of raw materials, a simple production process, and the ability to combine with chemical demulsifiers and reduce the effect on the environment [22]. However, it has several disadvantages, including the high cost of preparing enzymes and the stability of the emulsion, which greatly limit the use of the enzymatic method [23]. Therefore, a green, efficient, and cost-effective demulsification method needs to be developed urgently. Using fatty acids to demulsify emulsions can reduce costs and increase demulsification rate. Zhao et al. [24] used citric acid and oleic acid coupling for demulsification, and the demulsification rate was 95.87% under the optimal process condition. Gao et al. [4] demulsified peanuts with caproic acid and reported a demulsification rate of 97.64% under the optimum process conditions.

In our previous study, we found that fatty acids have good demulsification effect, especially heptanoic acid and octanoic acid [25]. In order to improve the demulsification rate of fatty acids and reduce the additive amount of fatty acids, two kinds of fatty acids were selected for complex demulsification. In this study, the optimum composite ratio of composite fatty acids (heptanoic acid and octanoic acid) was determined, and the process parameters were optimized. The differences on emulsion stability were subsequently investigated under optimal demulsification process, which was achieved by measuring the changes in emulsion particle size, potential, and conductivity before and after demulsification. The alterations in the constitution and configuration of emulsion proteins before and after demulsification were examined via sodium dodecyl sulfate-polyacrylamide gel electrophoresis (SDS-PAGE), liquid chromatography–mass spectrometry (LC–MS), and fluorescence spectroscopy. The microstructure of the emulsion was observed via confocal laser scanning microscopy (CLSM) before and after demulsification to determine the effects of fatty acids on the stability of the peanut emulsion.

## 2. Materials and Methods

### 2.1. Sample

The peanuts were obtained from the Henan Academy of Agricultural Sciences (Zhengzhou, China). Viscozyme^®^ was purchased from Novozymes Co., Ltd. (Shanghai, China). Heptanoic acid and octanoic acid were purchased from Shanghai Yuanye Biotechnology Co., Ltd. (Shanghai, China).

### 2.2. Preparation of Peanut Oil Emulsion

The oil body emulsion was made via AEE following the method described by Gao et al. [4], with slight modifications. Shelled peanuts were roasted in an oven at 50 °C for 12 h to remove the peanut skin. The treated peanuts (60 g) were soaked in deionized water and stored in a refrigerator (4 °C) for preparation. This mixture was crushed in a multifunctional food processor. Then, 1.5% plant hydrolase was added to the peanut pulp, and the mixture was stirred for 2 h in the 50 °C water bath and centrifuged at 5000 r/min for 15 min. The peanut emulsion in the upper layer was removed. The remaining aqueous and residual phases were centrifuged at 5000 rpm for 15 min. The peanut oil emulsion in the upper layer was removed again. The peanut emulsions were mixed.

### 2.3. Determination of the Oil Content in the Peanut Oil Mixture

The oil content of the emulsion was determined following the method described by Liu et al. [26]. Briefly, the chloroform–methanol solution (2:1, *v*/*v*) method was used for extracting oil in emulsions. The peanut oil mixture was combined with a solution of trichloromethane–methanol (2:1, *v*/*v*) at a 3:1 ratio. The samples were centrifuged at 5000 rpm for 10 min, after which the separated solids were subjected to the above process once more. Then, the extract was recovered and rotary evaporation was performed to remove the organic solvent, thus obtaining the oils and fats. The oil content of peanut emulsion was calculated using Equation (1).

(1) $$Oil\;content\;of\;emulsion\;(\%)=\frac{M_{2}-M_{1}}{M_{0}}\times 100$$

where M_0_ is the weight of emulsion (g); M_1_ is the weight of a round-bottomed flask (g); and M_2_ is the weight of a round-bottomed flask and oils (g).

### 2.4. Selection of the Composite Ratio of Composite Fatty Acids

Initially, 8 g of the emulsion was weighed, and the effects of different compound ratios on the demulsification rate were investigated. The assessment was conducted under the following conditions: material-to-liquid ratio of 1:4 (g/mL), reaction temperature of 50 °C, reaction time of 40 min, and fatty acid additive amount of 1.5%. Composite ratios of 4:1, 3:1, 2:1, 1:1, 1:2, 1:3, and 1:4 (heptanoic:octanoic acid) were explored. The peanut emulsion demulsification rate was calculated using Equation (2).

(2) $$Demulsification\;rate\;(\%)=\frac{W_{1}}{W_{0}\times \alpha }\times 100$$

where W_0_ is the weight of emulsion (g); W_1_ is the weight of free oil after demulsification (g); and α is the oil content of emulsion (%).

### 2.5. Optimization of Demulsification Conditions with Composite Fatty Acids

#### 2.5.1. The Effects of Different Conditions on Demulsification by Composite Fatty Acids

The effects of fatty acid addition (%), reaction time (min), reaction temperature (°C), and the solid-to-liquid ratio (*w*/*v*) on the demulsification rate of the oil body emulsion were investigated. The effect of fatty acid addition on demulsification was examined under the following conditions: 0.50%, 0.75%, 1.00%, 1.25%, and 1.50% fatty acid addition; a reaction temperature of 50 °C; a reaction time of 40 min; and a solid-to-liquid ratio of 1:4 (g/mL). Additionally, the effect of reaction time on demulsification was assessed under the following conditions: 1.0% fatty acid addition and a solid-to-liquid ratio of 1:4 (g/mL). Moreover, the effect of the reaction temperature on demulsification was evaluated under the following conditions: the addition of 1.0% fatty acid, a reaction time of 50 min, and temperatures of 30, 40, 50, 60, and 70 °C were investigated at a solid-to-liquid ratio of 1:4 (g/mL). The effect of the solid-to-liquid ratio on demulsification was examined under the following conditions: fatty acid addition content of 1.0%, a reaction time of 50 min, a reaction temperature of 60 °C, and solid-to-liquid ratios (*w*/*v*) of 1:1, 1:2, 1:3, 1:4, and 1:5.

#### 2.5.2. Orthogonal Optimization Tests

Following the methodology outlined in Section 2.5.1, single-factor experiments were conducted, and four factors were identified for inclusion in the *L*_9_ (3^4^) orthogonal optimization test: fatty acid addition, demulsification time, demulsification temperature, and solid-to-liquid ratio. The selected factors were grouped into three categories, with the demulsification rate serving as the test indicator to determine the optimal conditions for demulsification. The selected factors and classes are presented in Table 1.

### 2.6. The Particle Size, Zeta Potential, and Conductivity

First, 8 g of peanut emulsion was weighed, and three samples were prepared by adding heptanoic acid (YH37-G), octanoic acid (YH37-X), and a combination of heptanoic and octanoic acids (in a 1:3 ratio) (YH37-GX) to the peanut emulsion, under the following conditions: a solid-to-liquid ratio of 1:5, demulsification for 40 min at 70 °C. The control sample, YH37-KB, was prepared by adding deionized water to the peanut emulsion at a 1:5 ratio. Then, the mixed sample was introduced into the laser particle size analyzer (Baxter BT-9300H, Dandong Baxter Instrument Co., Ltd., Dandong, China). The refractive index of the peanut emulsion was selected as 1.45, and the mean particle size of the emulsion (D_4,3_) was determined. The zeta potential was measured by introducing the mixed liquid to the cuvette of the laser light scattering instrument (Zeta-sizer Nano ZSP, Marvin instrument Co., Ltd., Marvin, UK) and allowing it to reach equilibrium for 30 s.

### 2.7. The Sodium Dodecyl Sulfate-Polyacrylamide Gel Electrophoresis (SDS-PAGE)

Three demulsification samples and a blank control were prepared following the methodology described in Section 2.6. The samples were subsequently lyophilized. The effects of adding different fatty acids on the protein composition of the peanut emulsions were evaluated by SDS-PAGE. The method used was slightly modified from that described by Zhao et al. [24]. Briefly, a protein sample mixture (4 mg/mL) was prepared by adding sample buffer (2 mL Tris-HCl (0.5 mol/L, pH 6.8); 1 mL mercaptoethanol; 2 mL 10 g/100 g SDS; 0.5 mL 0.5 g/100 g bromophenolblue; 2 mL glycerinum; and 2.5 mL distilled water), and the protein was denatured by boiling in a water bath for 5 min. A 5% separating gel and a 12% concentrating gel were sequentially run, and different samples were added to the lane. After electrophoresis, the gel was fixed, stained with a staining solution (Coomassie brilliant blue R250, Yeasen Co., Ltd., Shanghai, China) for 2 h, and destained until the protein bands were prominent. Finally, the gel was washed with deionized water and photographed for analysis.

### 2.8. Fluorescence Spectroscopy

The samples prepared following the procedure outlined in Section 2.6 were weighed and combined with the prepared solution, which resulted in the formation of a solution that had a concentration of 0.5 mg/mL. Then, the endogenous fluorescence spectra of the samples were determined using a fluorescence spectrophotometer (FL970, Techcomp group, Co., Ltd. Shanghai, China). The parameters were set as follows: the excitation wavelength was 290 nm, the spectrum was scanned between 310 and 500 nm, and the excitation and emission slits were set at widths of 5 nm.

### 2.9. Observation of the Oil Body Emulsion Microstructure by CLSM

The changes of the microstructure of peanut emulsions during demulsification were determined via CLSM according to Li et al. [27]. Three demulsified samples and the control were prepared following Section 2.6. A total of 20 µL of 0.05% Fluorescein isothiocyanate (FITC, Macklin Co., Ltd., Shanghai China) and 50 µL of 0.02% Nile red (Yuanye Co., Ltd., Shanghai, China) were added and mixed, after which the sample was stained for 10 min. Next, 20 µL of the stained mixture was transferred to a slide and examined via CLSM (FV1000). The excitation and emission wavelengths were set as follows: FITC, 488 nm and 510–530 nm (green); and Nile Red, 545 nm and 570–620 nm (red).

### 2.10. Statistical Analysis

The experiments were carried out three times and the resulting data were expressed as the mean ± standard deviation. The data were processed and plotted using SPSS 27.0 and Origin 2022.

## 3. Results and Discussion

### 3.1. Determination of the Optimal Composite Ratio of Composite Fatty Acids

**Figure 1:**
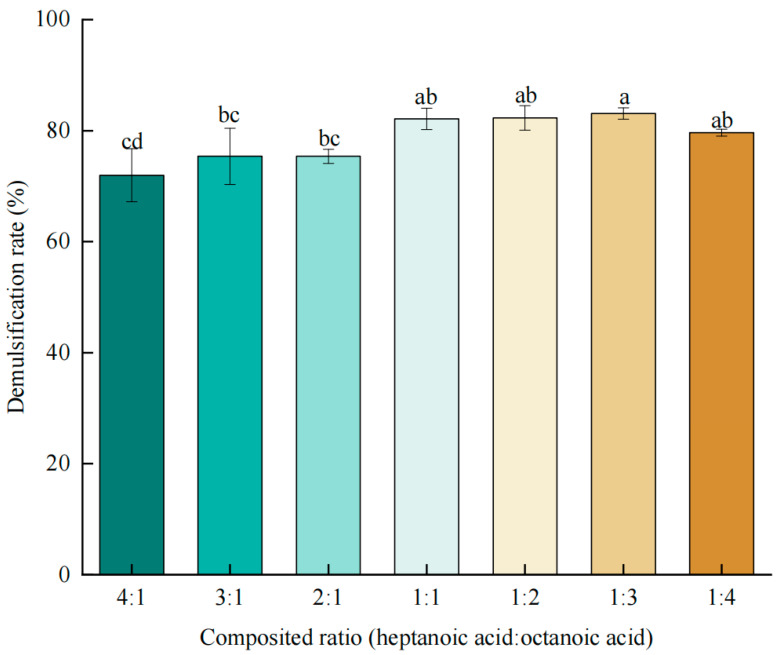
The effect of the composite ratio of heptanoic acid to octanoic acid on the demulsification rate. A significant difference between samples is indicated by different lowercase letters (*p* < 0.05).

Heptanoic and octanoic acids were proved to be useful in demulsification of oil body emulsions [25]. In this study, a combination of the two types of fatty acids was used to demulsify. The effect of the composite ratio of heptanoic acid to octanoic acid on the demulsification rate is illustrated in Figure 1. When the ratio was changed, the demulsification rate of the peanut emulsion increased. When the ratio of heptanoic acid to caprylic acid was 1:1, there was no significant change. Although the demulsification rate increased gradually with the increase in the ratio of caprylic acid, there was no significant difference. The ratio of 1:3 (heptanoic acid:caprylic acid) for the composite fatty acids was determined and used in the next study.

### 3.2. Optimization of Complex Demulsification of Heptanoic Acid and Octanoic Acid

#### 3.2.1. Effects of Fatty Acid Addition on the Demulsification of Peanut Emulsions

**Figure 2:**
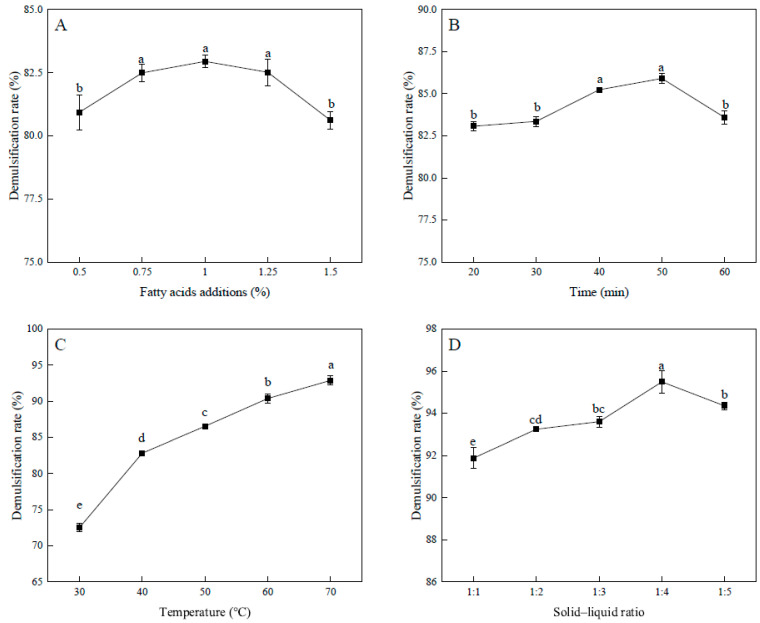
Effect of different conditions on demulsification rates of peanut emulsion. (**A**) The addition of composite fatty acids, (**B**) reaction time, (**C**) reaction temperature, and (**D**) solid–liquid ratio. A significant difference between samples is indicated by different lowercase letters (*p* < 0.05).

The effect of adding composite fatty acids on the demulsification process is shown in Figure 2A. The demulsification rate of the peanut emulsion increased significant (*p* < 0.05) when the addition of composite fatty acids from 0.5 to 0.75%. The demulsification rate also increased gradually when the addition amount continued to increase (0.75~1.25%), but there was no significant difference (*p* > 0.05). The demulsification rate reached its highest point (82.95 ± 0.25%) when the addition amount was 1.00%. The initial pH of the peanut emulsion was about 6.8, which rendered the emulsion more stable and less susceptible to demulsification. As the pH decreased to the pI, the stability of the emulsion decreased [28]. The addition of fatty acids increased the pH of the emulsion close to the pI, which led to demulsification. The addition of about 1% fatty acids resulted in the pH close to the pI (pH 4.5). The zeta potential of the peanut emulsion was close to 0. The oil droplets clumped together because there was no more electrostatic repulsion. Finally, the addition of composite fatty acids (0.5~1%) were added for orthogonal optimization.

#### 3.2.2. Effect of the Reaction Time on the Demulsification of Peanut Emulsions

The effect of the reaction time on the demulsification rate of the emulsion is illustrated in Figure 2B. As the reaction time increased, the demulsification rate of the peanut emulsion initially increased, then decreased, and subsequently plateaued after 40 min. When the reaction time was 50 min, the demulsification rate of the emulsion was 85.91 ± 0.31%. Owing to the low density of heptanoic acid, which is slightly soluble in water, it tended to be located in the upper layer of the emulsion. Fatty acids take time to diffuse into the oil droplets, but too much time leads to the decomposition of the oil [29], resulting in the loss of a lot of energy. The demulsification rates of the reaction time for 40 min and 50 min were not significantly different (*p* > 0.05). Thus, a reaction time range of 40 to 60 min was chosen for orthogonal optimization.

#### 3.2.3. Effect of Reaction Temperature on the Demulsification of Peanut Emulsions

The effect of temperature on the demulsification rate of the emulsion is illustrated in Figure 2C. The results indicated that the demulsification rate of peanut emulsions increased significantly (*p* < 0.05) from 72.51 ± 0.59% to 92.91 ± 0.64% with an increasing in the temperature. As the temperature increased, the fatty acids spread faster through the system, the oil droplets moved more, and the molecular interface membrane composed of phospholipids and proteins was unfolded. This increased the contact area with the emulsifier and improved the demulsification rate [3]. Considering that excessively high temperatures consumed a large amount of energy and could also accelerate the oxidation of oils, affecting their quality, the reaction temperature of 50~70 °C was selected for orthogonal optimization.

#### 3.2.4. Effect of the Solid–Liquid Ratio on the Demulsification of Peanut Emulsions

The effect of the solid–liquid ratio on the demulsification rate is shown in Figure 2D. As the solid–liquid ratio increased, the demulsification rate of the peanut emulsion initially increased and then decreased. The maximum demulsification rate was 95.88 ± 0.54% at a solid–liquid ratio of 1:4. Insufficient deionized water in the system would lead to an increase in viscosity, which impeded the requisite contact between the emulsion and fatty acids [4]. The demulsification rate decreased when excess deionized water was present. This occurred probably because the oil droplets were diluted, which hindered the polymerization process. Thus, the solid–liquid ratio of 1:3~1:5 was selected for orthogonal optimization.

#### 3.2.5. Results of Orthogonal Optimization of Demulsification by Composite Fatty Acids

According to the result of single-factor experiments, the fatty acid addition (0.5~1%), demulsification time (30~50 min), demulsification temperature (50~70 °C), and solid-to-liquid ratio (1:3~1:5) were used for orthogonal optimization. The results were evaluated and are shown in Table 2. The value of R (range) revealed the effects of the four factors on the demulsification rate in the following order: A > C > B > D, i.e., addition of composite fatty acids > temperature > time > solid–liquid ratio. The optimal condition of demulsification was A_2_B_2_C_3_D_3_. The four factors significantly affected the demulsification rate (Table 3). The optimal condition for demulsification by composite fatty acids was as follows: 1.00% composite fatty acids addition, demulsification for 40 min at 70 °C, and a 1:5 solid–liquid ratio. The optimal demulsification rate was 97.95% ± 0.03% under these condition.

### 3.3. Effects of the Demulsification of Composite Fatty Acids on the Physicochemical Properties of Emulsions

#### 3.3.1. Effects of Composite Fatty Acid Demulsification on Emulsion Particle Size

**Figure 3:**
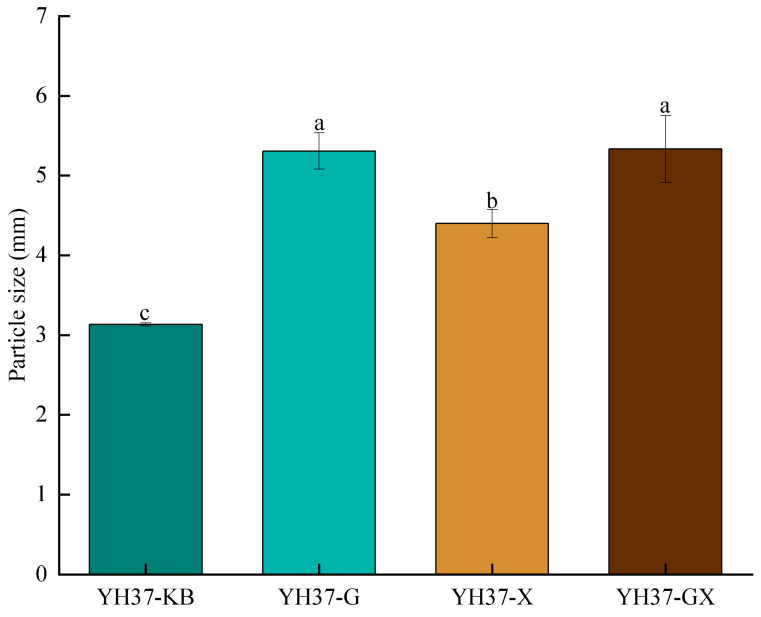
The effect of different fatty acids added on different size particles of peanut emulsion. A significant difference between samples is indicated by different lowercase letters (*p* < 0.05).

Particle size and potential are important parameters for assessing the stability of peanut emulsions [30,31,32]. The smaller the particle size, the more stable the emulsion [33]. The effects of adding different fatty acids on different size particles of the emulsions are shown in Figure 3. Compared to the original emulsions, all three samples with added fatty acids presented an increase in particle size. This finding was consistent with the results reported by Zheng et al. [34], who reported that adding medium-chain fatty acids increases the particle size of emulsions. The particle size of YH37-G was greater than that of YH23-X, whereas the particle size of YH37-GX was greater than those of YH37-KB and YH37-X. Fatty acids could intensify the Brownian motion between emulsions, decrease the electrostatic repulsion between droplets adsorbed by protein particles, and increase the propensity for droplet instability. This leads to the aggregation of droplets, an increase in droplet volume, and an increase in the particle size of the emulsion [35,36]. Compared to octanoic acid, composite fatty acids and heptanoic acid exhibited the most effective demulsification properties.

#### 3.3.2. Effects of Composite Fatty Acids on Emulsion Potentials During Demulsification

**Figure 4:**
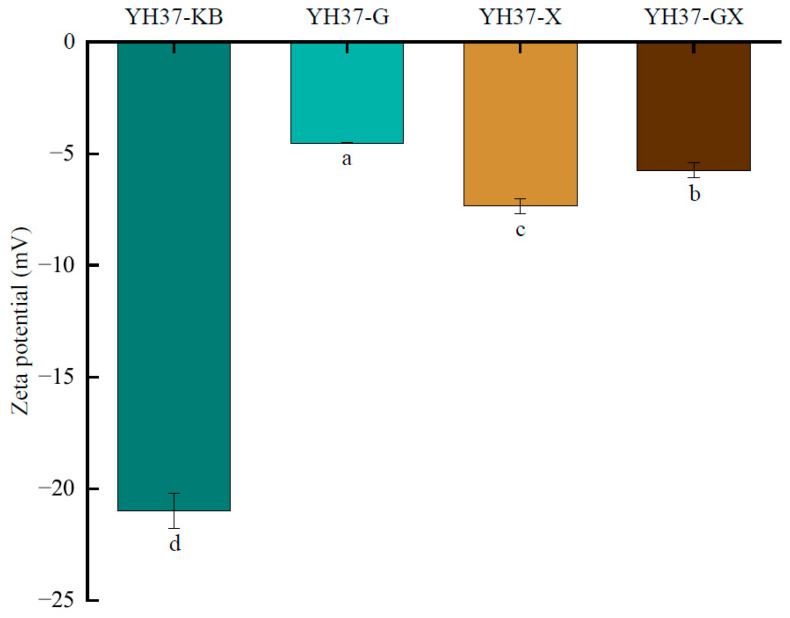
The effect of different fatty acids addition on the zeta potential of peanut emulsion. A significant difference between samples is indicated by different lowercase letters (*p* < 0.05).

The absolute zeta potential can effectively reflect the electrostatic interaction between fat globules. A larger zeta potential indicates a greater net surface charge, which results in an increase in electrostatic repulsion between fat globules. This reduces the probability of water precipitation and agglomeration, thus enhancing the stability of the emulsion system [30,31]. The effect of distinct fatty acid additions on the emulsion potential is illustrated in Figure 4. As illustrated in the figure, the absolute zeta potentials of the samples demulsified with the addition of fatty acids were lower than those of the original peanut emulsion. The absolute zeta potentials could be arranged in ascending order as follows: YH37-G, YH37-GX, YH37-X, and YH37-KB. This finding indicated that the addition of fatty acids caused the peanut emulsion to deviate from its original pI, reducing the electrostatic repulsion between oil droplets and causing oil droplet aggregation in the emulsion. On the other hand, it was possible that heptanoic acid could more effectively change the pH of the emulsion than octanoic acid. Meanwhile, the increase in temperature during demulsification might lead to a change in zeta potential.

#### 3.3.3. Changes of the Emulsion Conductivity During Demulsification by Composite Fatty Acids

**Figure 5:**
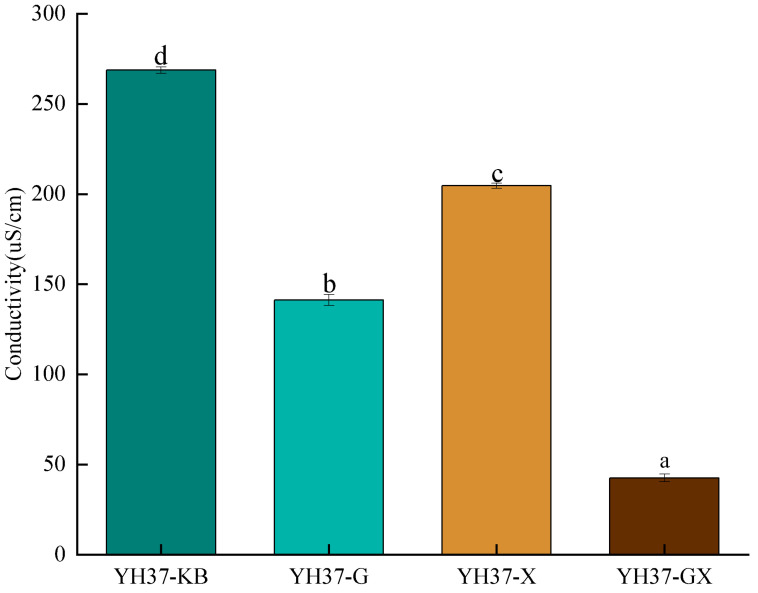
The effect of different fatty acid additions on the conductivity of peanut emulsion. A significant difference between samples is indicated by different lowercase letters (*p* < 0.05).

The effect of distinct fatty acid additions on the conductivity of the emulsion is illustrated in Figure 5. The conductivity of an emulsion can be used to ascertain the specific type of emulsion present [37]. Zhao et al. [24] found that the conductivity of the emulsion began to decrease significantly with the increase in oleic acid addition, and the conductivity decreased from 114.5 μS/cm to less than 1 μS/cm, which was consistent with this result. When the conductivity is high, water acts as a continuous phase, and the emulsion exhibits the oil-in-water (O/W) type; when the conductivity of the emulsion is low, the oil is continuous, and the emulsion is the water-in-oil (W/O) type [38]. Compared to the initial peanut emulsion, the addition of fatty acids to the demulsified samples reduced conductivity considerably. The conductivity was found to decrease in the following order: YH37-GX < YH37-G < YH37-X < YH37-KB. The conductivity of the initial emulsion was 270.6 µS/cm and the emulsion was the O/W type. The conductivity of YH37-GX was significantly lower than that of YH37-KB, and the emulsion probably underwent a phase transition from O/W to W/O.

### 3.4. Effects of the Demulsification by Composite Fatty Acids on Proteins in Emulsions

#### 3.4.1. Effects of the Demulsification by Composite Fatty Acids on the Protein Composition of Emulsions

**Figure 6:**
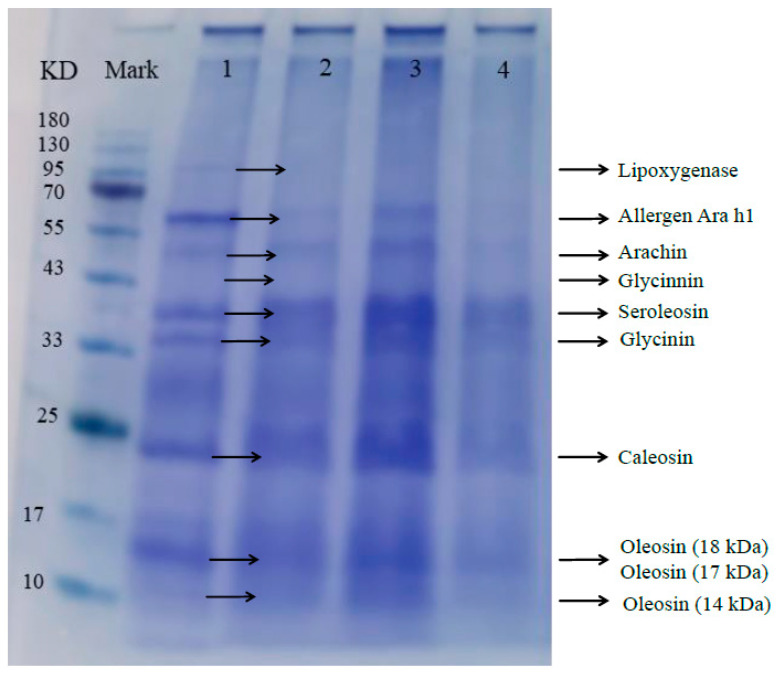
The results of SDS-PAGE and protein compositions identified before and after demulsification by different fatty acids. Lane 1: protein compositions in original oil body emulsion. Lane 2–4: protein compositions of the demulsification samples with the addition of heptanoic acid (YH37-G), octanoic acid (YH37-X), and composite fatty acids (YH37-GX), respectively.

The stability of the emulsion related to the type and structure of the proteins on the oil–water interface [39]. Dias et al. [40] found that enzyme treatment can reduce the molecular mass of protein by hydrolyzing interface protein, effectively destroy the structure of interface protein, and destroy the integrity of interface film, thus promoting the aggregation of oil droplets. The SDS-PAGE analysis of the peanut emulsion proteins is shown in Figure 6. As illustrated in the figure, the protein species and content of the demulsified samples by fatty acids were lower than those of the original peanut emulsion. Kim et al. [41] found that protein concentrations in all samples decreased after medium-chain fatty acid treatment. Some bands of proteins with larger molecular weights (lipoxygenase) were no longer visible in the emulsion. Compared to YH37-X, the bands of YH37-G were lighter, and those of YH37-GX were the lightest. Compared to heptanoic or octanoic acids, the sample of composite fatty acids reduced the interfacial protein content to a greater extent, and the coverage of interfacial proteins decreased. This might be because different fatty acids have different ways and positions of action on proteins, and the integrity of protein interfacial membranes was damaged more seriously than that of a single fatty acid.

#### 3.4.2. Changes in the Tertiary Structure of Emulsion Proteins During Demulsification Using Composite Fatty Acids

**Figure 7:**
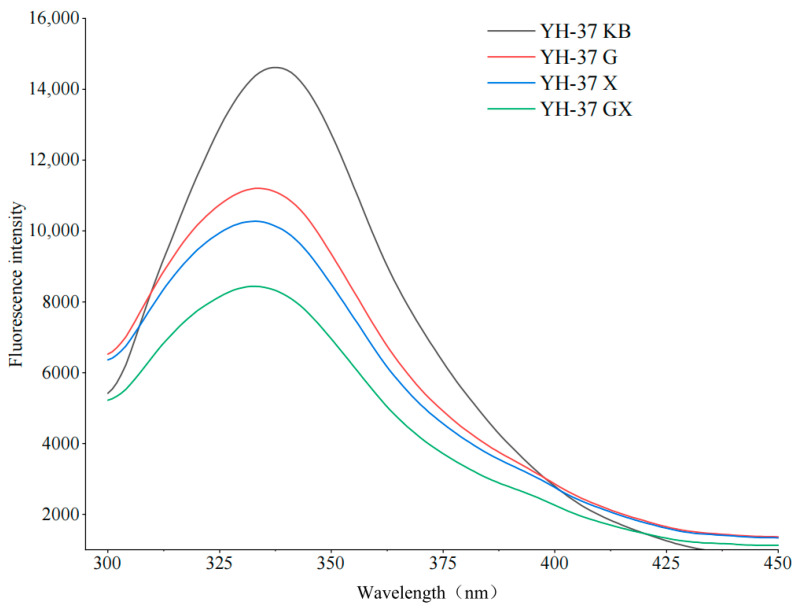
The fluorescence spectra of the demulsification samples with the addition of different fatty acids.

Fluorescence spectroscopy was used to illustrate the evolving patterns of tertiary structures of proteins in peanut emulsions. As a consequence of proteins containing amino acid residues that could absorb and emit fluorescence, the degree of alteration in tryptophan residue served as a reflection of structural changes occurring within the protein [42,43]. Compared to the control, the fluorescence intensities of the samples demulsified by fatty acids were lower in Figure 7. The sample with the addition of the composite fatty acids presented the lowest fluorescence intensity. This phenomenon might be attributed to the interaction between amino acid residues within the emulsion system and fatty acids. Additionally, introducing fatty acids might alter the surrounding microenvironment of the system. Compared to the control, the demulsified samples with added fatty acids underwent a ‘blueshift’. These findings indicated that when fatty acids demulsify the peanut emulsion, the tryptophan residues were buried within the protein, resulting in a shift from a polar to a nonpolar environment [44].

### 3.5. Effects of the Demulsification of Composite Fatty Acids on the Microstructure of Emulsions

**Figure 8:**
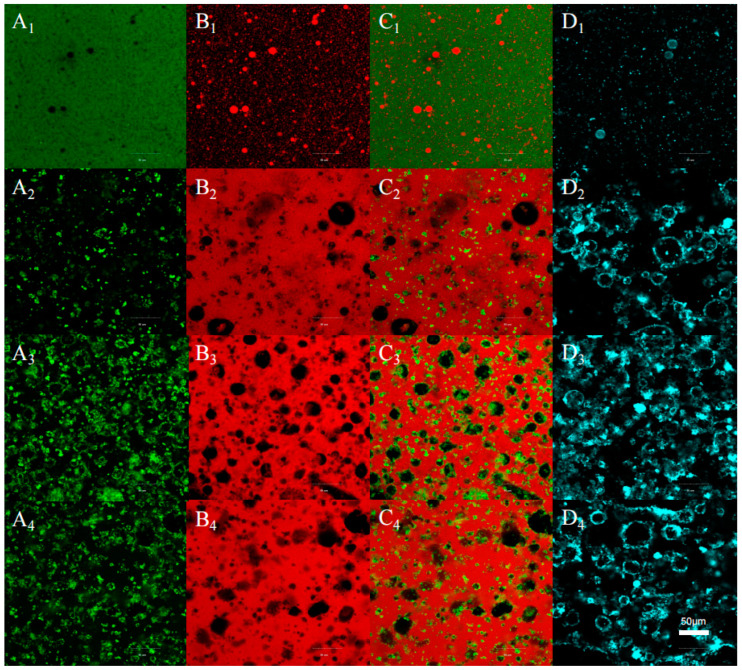
CLSM of the emulsion microstructure before and after demulsification. (**A**) Proteins stained by FTIC (green), (**B**) oils stained by Nile red (red), (**C**) combination of proteins and oils, and (**D**) phospholipids stained by B-DHPE (blue). (**1**) Original peanut emulsion, (**2**) the emulsion demulsified by heptanoic acid, (**3**) the emulsion demulsified by octanoic acid, and (**4**) the emulsion demulsified by composite fatty acids. Note: (**A**) protein staining; (**B**) lipid staining; (**C**) combination diagram; and (**D**) phospholipid staining. (**1**) Original peanut emulsion; (**2**) addition of heptanoic acid demulsification; (**3**) addition of caprylic acid demulsification; and (**4**) composite fatty acid demulsification.

As illustrated in Figure 8, CLSM was performed to assess the alterations in the microstructure of the emulsion under the conditions of demulsification with the addition of different fatty acids. The oils, proteins, and phospholipids in the emulsion were stained with Nile red, FITC, and B-DHPE, respectively, which emitted red, green, and blue fluorescence under irradiation with different excitation wavelengths. The distributions of oil and protein in the system were largely similar to those in the initial emulsion, which served as a control. As shown in Figure 8C_1_, most of the oil droplets were spherical, with phospholipids forming rings distributed on the surfaces of the oil droplets. Meanwhile, proteins wrapped around the oils, resulting in the O/W emulsion due to the interaction of phospholipids and proteins. The addition of fatty acids resulted in the formation of several green aggregates and a substantial red area. This finding indicated that the interfacial membrane, which was formed by phospholipids and proteins, was disrupted, leading to the aggregation of the oil droplets. Decker et al. [45] showed that fatty acids could regulate the pH of the emulsion system, thereby destroying the interfacial membrane that was formed by phospholipids and proteins. After demulsification, red and blue rings formed in the system (Figure 8C,D). This indicated that the proteins were degraded and the products were dispersed in the aqueous phase and wrapped in the continuous oil phase, resulting in the formation of localized W/O structures. Moreover, the phospholipids formed rings that covered the surface of the oil.

### 3.6. Proposed Demulsification Mechanism of Composite Fatty Acids

**Figure 9:**
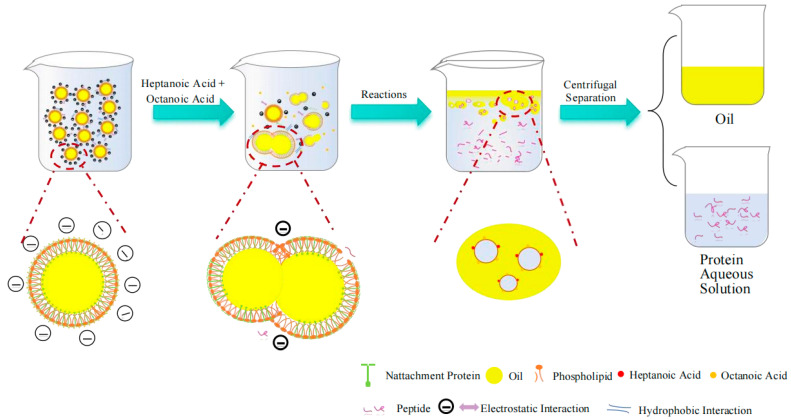
The presumed mechanism of demulsification using composite fatty acids.

Based on these results, the mechanism for the effective demulsification of composite fatty acids was proposed, as illustrated in Figure 9. In the initial emulsion, phospholipids and proteins formed the interfacial membrane around the oil droplets. The emulsion was stable because there were more negative charges, and it exhibited the oil-in-water emulsion. Upon the addition of composite fatty acids to the emulsion, fatty acids interacted with phospholipids and the structures of proteins changed [44], the phospholipid-protein membrane was damaged. The proteins fragments were dispersed in the water phase. The addition of composite fatty acids could reduce the absolute potential, thereby weakening electrostatic interactions. This would lead to aggregation between oil droplets, forming larger-sized oil droplets, thus achieving demulsification.

## 4. Conclusions

This study explored a novel demulsification method using composite fatty acids during aqueous enzymatic extraction. Under the optimal condition, the ratio of heptanoic acid and octanoic acid of 1:3, the fatty acid addition of 1.00%, demulsification time of 40 min, the demulsification temperature of 70 °C, and the solid–liquid ratio of 1:5, the demulsification rate was 97.95 ± 0.03%. This study provides a new way to realize efficient demulsification. This ratio increased the demulsification rate while simultaneously reducing costs. The properties of emulsion were altered after demulsification using composite fatty acids, such as increasing the particle size of the oil bodies, decreasing the absolute zeta potential, and lowering conductivity. Moreover, this study demonstrated that heptanoic acid exhibits greater efficacy than octanoic acid. The effect of demulsification followed the order: heptanoic and octanoic acid, heptanoic acid, and octanoic acid. Above all, this study sheds light on the mechanisms of emulsion destabilization from studying the changes in protein structure, interfacial properties, and emulsion type transformation (from O/W to W/O). This approach introduces a new and effective strategy for demulsification, addressing the challenge of stable O/W emulsions that hinder oil extraction. It provides a practical and efficient alternative to traditional methods.

## Figures and Tables

**Table 1:** The selected factor levels of demulsification for peanut emulsion.

Factor Levels	Independent Variables			
	Fatty Acid Addition (A)/%	Demulsification Time (B)/Min	Demulsification Temperature (C)/°C	Solid–Liquid Ratio (D)/(g/mL)
1	0.75	30	50	1:3
2	1.00	40	60	1:4
3	1.25	50	70	1:5

**Table 2:** Results of orthogonal tests of demulsification of peanut emulsion.

No.	A	B	C	D	Y/%
1	1	1	1	1	82.37 ± 0.08
2	1	2	2	2	92.93 ± 0.71
3	1	3	3	3	88.83 ± 0.57
4	2	1	2	3	96.78 ± 0.25
5	2	2	3	1	96.03 ± 0.03
6	2	3	1	2	92.30 ± 0.19
7	3	1	3	2	91.67 ± 1.39
8	3	2	1	3	91.77 ± 0.83
9	3	3	2	1	94.73 ± 0.40
k_1_	88.04	90.27	88.81	91.04	
k_2_	95.04	93.58	92.18	92.30	
k_3_	92.72	91.95	94.81	92.46	
R	4.68	1.68	3.36	1.26	
A > C > B > D					

**Table 3:** Analysis of variance of the results of the demulsification rate test.

Source of Variance	Sum of Squared Deviations	Degrees of Freedom	Mean Square	F-Value	Significance
A	76.1685	2	38.0845	92.059	**
B	16.3695	2	8.1845	19.784	**
C	54.2065	2	27.1030	65.515	**
D	3.603	2	1.8015	4.3545	**

Note: ** indicates a highly significant difference (*p* < 0.01).

## Data Availability

The original contributions presented in this study are included in the article. Further inquiries can be directed to the corresponding authors.
